# Avalanche photodiodes with multiple multiplication layers for coherent detection

**DOI:** 10.1038/s41598-022-21041-6

**Published:** 2022-10-03

**Authors:** Zohauddin Ahmad, Po-Shun Wang, Naseem, Yu-Cyuan Huang, Yan-Chieh Chang, You-Chia Chang, Yi-Shan Lee, Jin-Wei Shi

**Affiliations:** 1 Department of Electrical Engineering, National Central University, Taoyuan, 320 Taiwan; 2 Department of Photonics, National Yang Ming Chiao-Tung University, Hsinchu, 300 Taiwan

**Keywords:** Electrical and electronic engineering, Optics and photonics

## Abstract

We demonstrate a novel avalanche photodiode (APD) design which fundamentally relaxes the trade-off between responsivity and saturation-current performance at receiver end in coherent system. Our triple In_0.52_Al_0.48_As based multiplication (M-) layers with a stepped electric (E-) field inside has more pronounced avalanche process with significantly less effective critical-field than the dual M-layer. Reduced E-field in active M-layers ensures stronger E-field allocation to the thick absorption-layer with a smaller breakdown voltage (V_br_) resulting in less serious space-charge screening effect, less device heating at high output photocurrent. Compared to the dual M-layer reference sample, the demonstrated APD exhibits lower punch-through (− 9 vs. − 24 V)/breakdown voltages (− 43 vs. − 51 V), higher responsivity (19.6 vs. 13.5 A/W), higher maximum gain (230 vs. 130), and higher 1-dB saturation-current (> 5.6 vs. 2.5 mA) under 0.95 V_br_ operation. Extremely high saturation-current (> 14.6 mA), high responsivity (7.3 A/W), and decent O-E bandwidth (1.4 GHz) can be simultaneously achieved using the demonstrated APD with a 200 µm active window diameter. In coherent FMCW LiDAR test bed, this novel APD exhibits a larger signal-to-noise ratio and high-quality 3-D images than the reference dual M-layer and high-performance commercial p-i-n PD modules, while requiring significantly less optical local-oscillator (LO) power (0.5 vs 4 mW).

## Introduction

Avalanche photodiodes (APD) have come to play an important role at the receiving end in several different applications, such as fiber communications^1,2^, biosensing^3^, LiDAR^4–7^, quantum photonics^8^, quantum computing^9,10^ and optical wireless communications^11^, over the last few decades. Compared with the other semiconductor-based photodetectors with their large internal gain, which include phototransistors^12^ and photoconductors^13^, APDs usually allow shorter internal response times, wider optical-to-electrical (O-E) bandwidths, lower noise-equivalent-power (NEP) ratios, and higher sensitivity performance. On the other hand, in contrast to their p-i-n PD counterparts with their high unit gain, the penalty for high gain in the APD, due to the additional carrier multiplication process which occurs inside its active layers, is its lower output saturation current density and smaller O-E bandwidth. Recently, there has been strong demand for photoreceivers with a larger dynamic range for use in coherent systems. It has thus become important to produce APD devices which can sustain high responsivity and high-speed performance under a high saturation output current^14–16^. Take the coherent receivers in the frequency modulated continuous-wave (FMCW) LiDAR^17^ for example. A highly linear p-i-n photodiode, which can deliver a high saturation RF output power under strong optical local oscillator (LO) power pumping, is highly desired to amplify the weak received light. However, significant optical insertion loss remains a challenge in the advanced photonic integrated circuit (PIC)-based FMCW LiDAR system, limiting the output optical LO power (to several mWs)^18–20^. Vertical-illumination avalanche photodiodes (APDs) can provide the large optical window to minimize the coupling loss of the weak reflected light from free space to the APD and its additional multiplication gain is also needed to reduce the optical power budget for coherent detection. In our previous work, we provided a proof-of-concept demonstration that a large-area APD (200 µm diameter optical window) would exhibit superior performances to that of a p-i-n PD in an FMCW LiDAR system^21,22^.

In this work, we demonstrate a novel In_0.52_Al_0.48_As based vertically-illumination APD designed to further overcome the fundamental trade-off between the output saturation current density and multiplication gain. In the traditional APD, with high-responsivity performance, its saturation current is usually limited by the space-charge screening (SCS) effect in the thick In_0.53_Ga_0.47_As absorption layer (~ 2 µm)^14^. By lowering the doping density in the charge layer, a stronger electric (E-) field can be allocated to the absorption layer to suppress the SCS effect. However, this approach always leads to an increase in the breakdown (operation) voltage (V_br_) and more serious device heating under high-power operation. Here, by the adoption of several (triple) In_0.52_Al_0.48_As based multiplication (M-) layers with a stepped electric (E-) field inside, the avalanche process becomes more pronounced, and we can effectively reduce the required critical field ensuring the allocation of a stronger E-field within the thick absorption layer under a smaller V_br_ for APD operations. Compared with the dual M-layer reference sample, the demonstrated APD, with its close absorption and multiplication layer thickness values, exhibits both smaller punch-through (V_pt_) (− 9 vs. − 24 V)/ V_br_ (− 43 vs. − 51 V) voltages, higher responsivity (19.6 vs. 13.5 A/W), larger maximum gain (230 vs. 130), and higher 1-dB saturation current (> 5.6 vs. 2.5 mA) under 0.95 V_br_ operation. In the coherent FMCW LiDAR test bed, this novel design exhibits a larger signal-to-noise ratio in each pixel and much better quality of constructed 3-D images than those obtainable using the reference dual M-layer sample or high-performance commercial p-i-n PD modules, with much less optical local-oscillator (LO) power required (0.5 vs. 4 mW). This novel APD structure offers a new way to further enhance the sensitivity of the next generation coherent communication and FMCW LiDAR systems.

### Device structure and fabrication

**Figure 1:**
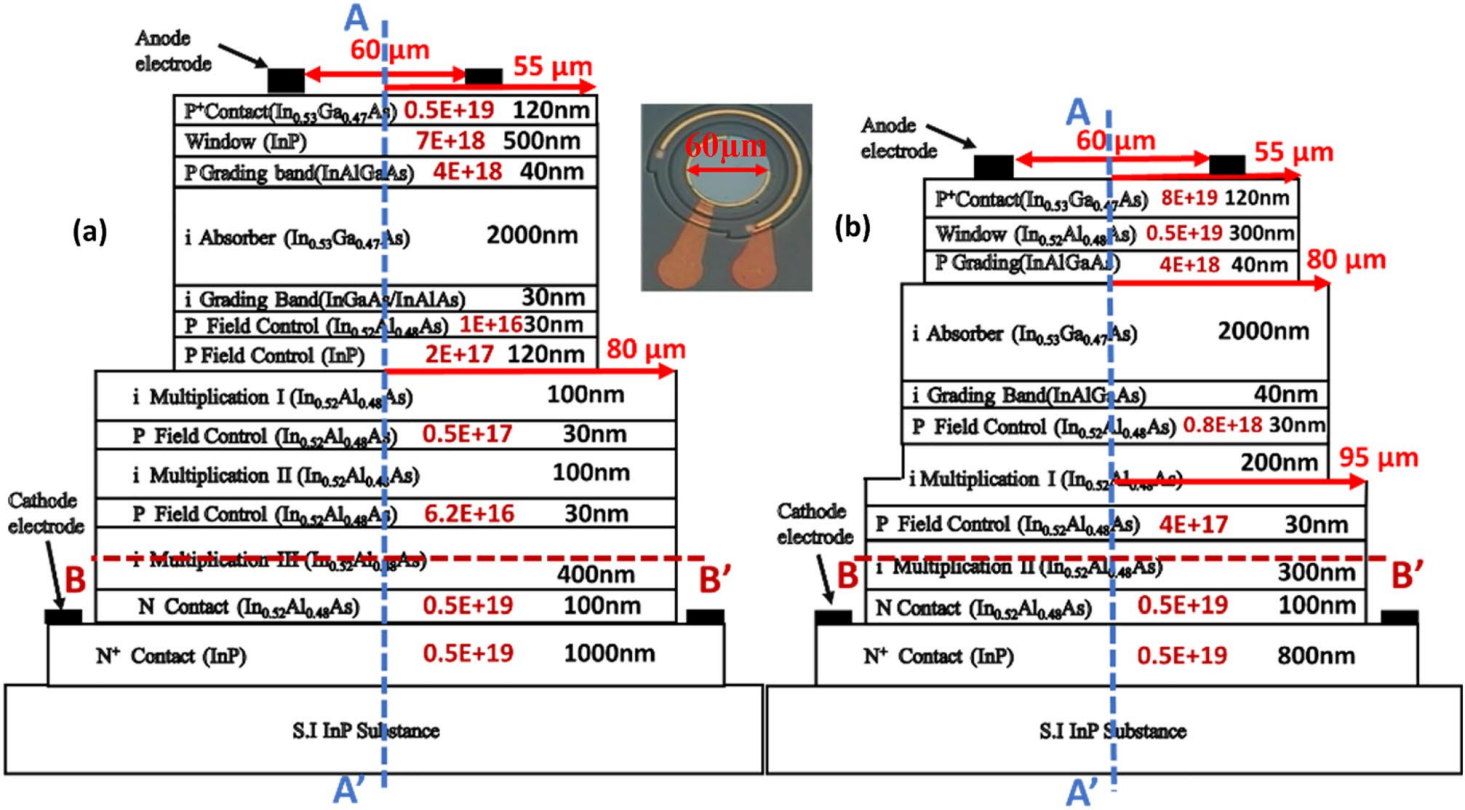
Cross-sectional view of the demonstrated APDs. (**a**) Triple M layer (Device A). (**b**) Dual M layer (Device B). Inset shows the top view with an active window diameter of 60 µm in both cases. The thickness and doping density (cm^−3^) of each layer have been specified.

**Figure 2:**
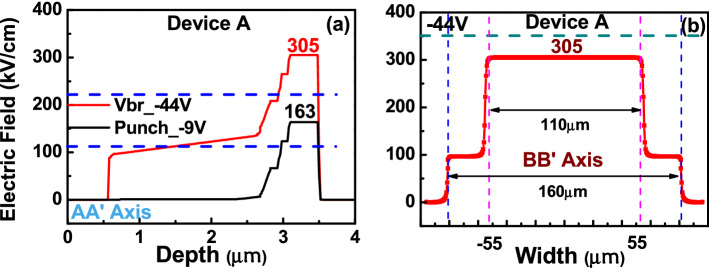
Electric field profiles at V_pt_ and V_br_ for device A along the directions. (**a**) AA′. (**b**) BB′.

**Figure 3:**
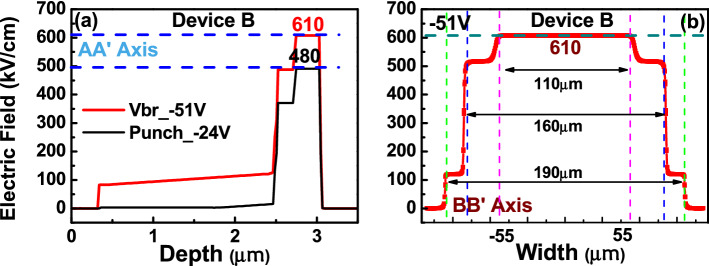
Electric field profiles at V_pt_ and V_br_ for device B along the directions. (**a**) AA′. (**b**) BB′.

**Figure 4:**
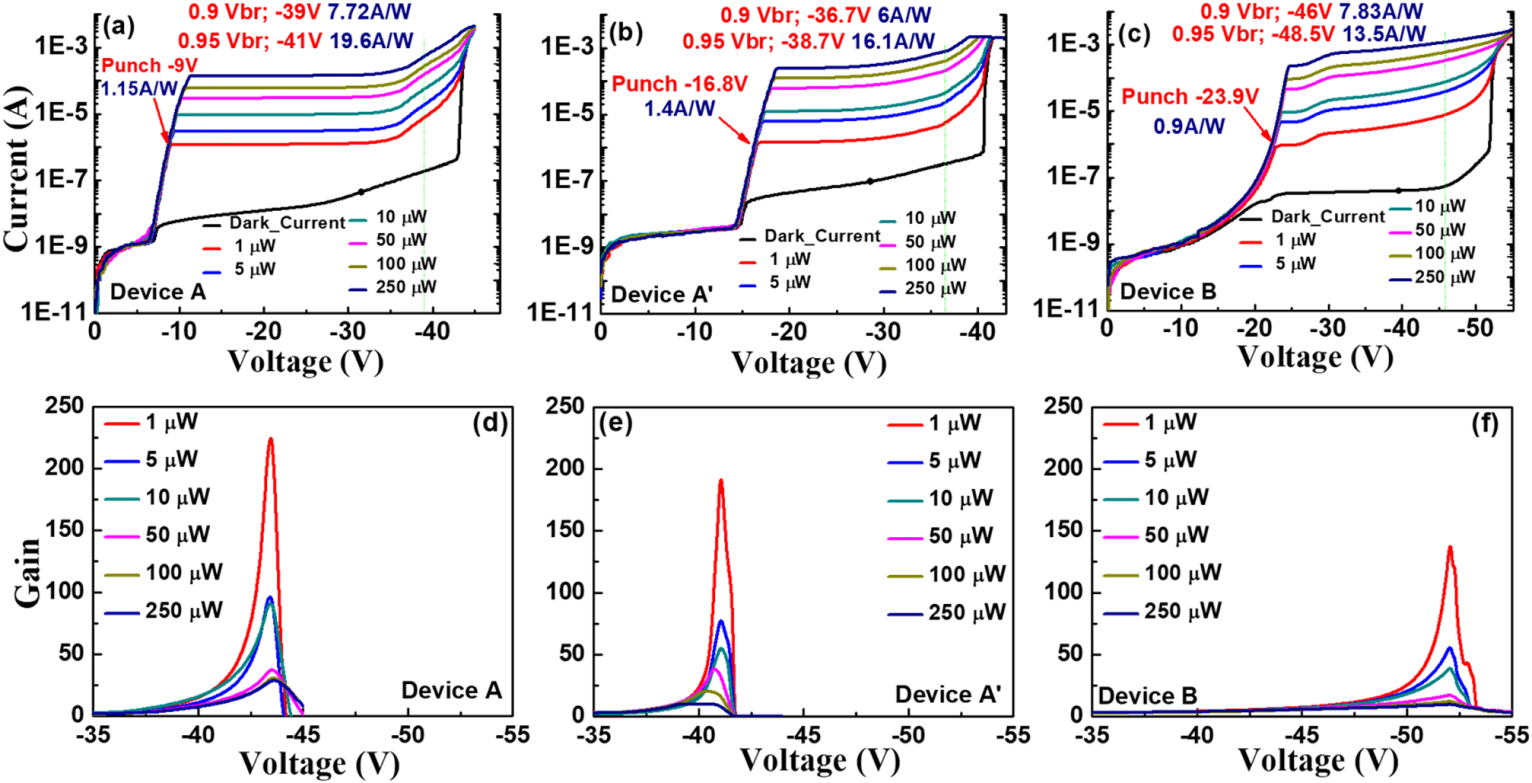
(**a**–**c**) shows measured bias dependent dark current and photocurrent (**d**–**f**) shows operation gain versus bias voltage under different optical pumping powers for Device A, Device A′ and Device B, respectively.

The core of this paper is to study the influence of different number of M- (charge) layer inside APD on its dynamic and static performances. Here, two kinds of APDs structures with triple (device A) and dual (device B^21^) M-layers were demonstrated and investigated. Figure 1a,b depicts conceptual cross-sectional view of these two APD mesa structures. Note that, for simplicity, Fig. 1 is not drawn to scale. The top view of the fabricated device with an active window (mesa) diameter of 60 (110) µm is shown in the inset to Fig. 1. From top to bottom, the structure of device B is composed of a p +-In_0.53_Ga_0.47_As contact layer, p +-In_0.52_Al_0.48_As window layer, intrinsic In_0.53_Ga_0.47_As absorbing layer, two p-type In_0.52_Al_0.48_As charge (field control) layers, and two intrinsic In_0.52_Al_0.48_As multiplication layers and N + In_0.52_Al_0.48_As/InP contact layers. At the interfaces between the absorber/window and absorber/multiplication layers, two In_0.52_Al_x_Ga_0.48-x_As graded bandgap layers (GBLs) are introduced. The thickness and the doping concentration of each epi-layer have both been specified in Fig. 1. As can be seen, both devices have the same In_0.53_Ga_0.47_As absorption layer thickness as 2 µm and close M-layer thickness values (600 (A) vs. 500 nm (B)). Although the thicknesses of other layers, such as, contact, window, and GBLs in both these two devices are not exactly the same, these layers usually don’t play vital roles in the dynamic and static performances of APDs and thus don’t affect the purpose of this work, i.e., to study the influence of different number of M-layers on APD performances. Compared with the traditional APD with a single M-layer inside, the M-region in A and B has been subdivided into different parts using additional charge control layers^14,21,22^, which results in a stepped electric field profile in the M-region. In device A, for example, the 600 nm thick multiplication (M-) layer is subdivided into three parts, 100, 100, and 400 nm in thickness. The multiple M-layer design of our APD structure restricts the multiplication process to a thin high-field region effectively rather than the whole thickness of the M-layer, thus avoiding an increase in the tunneling dark current and directly shrinking the thickness of the M-layer in the APD. Furthermore, at high-gain operation the cascade avalanche process in our demonstrated multiple M-layer structure restricts driving of single M-layer into the deep avalanche region. Overall, a shorter avalanche delay time and a larger GBP can be achieved using the same M-layer thickness when compared to traditional APD design as demonstrated in our previous work^21,22^. Silvaco Technology Computer Aided Design (TCAD) tools are used to simulate the electric field distribution within the device. Figures 2 and 3 show the calculated electric fields for our demonstrated APD along the vertical (AA′) and horizontal (BB’) directions, as shown in Fig. 1, at the punch-through (V_pt_) and breakdown (V_br_) voltage. The measured values of V_br_ and V_pt_ will be discussed in detail later in the measured I–V curves as shown in Fig. 4. As we can see, even under V_br_ operation, the E-field in the In_0.53_Ga_0.47_As layer is much below its critical field (~ 150 kV/cm^23^), for both devices (A and B). Moreover, as can be seen in device A, the effective critical field in In_0.52_Al_0.48_As M-layer can be lowered to around 300 kV/cm, due to fact that the cascade avalanche process in our M-region results in a more pronounced impact ionization process under a lower E-field. The reported critical field in the In_0.52_Al_0.48_As M-layer is at around 600 kV/cm^24^. During the design of an APD structure, a lower critical field implies a smaller operating voltage (V_br_), less serious device heating, and the stronger E-field allocated to the thick In_0.53_Ga_0.47_As absorber layer can suppress the SCS effect leading to a higher saturation output photocurrent density. Overall, our multiple M-layer design allows a fundamental relaxation of the limitation on the saturation of a high gain APD and provides a shorter avalanche delay time and a larger GBP is achieved than that of traditional APD design having same M-layer thickness.

As shown in Fig. 1, in order to suppress the edge breakdown at the edge of the bottommost M-layer, which has the strongest E-field, both devices adopt the etched mesa structure with the same working principle. Here, we etch through the field control (charge) layers, stopping at the M-layer in both devices (A and B)^14,21,25^, which can nearly zero the E-field in the edge of M-layer. Besides, in device A, a composite charge layer (In_0.52_Al_0.48_As/InP) design is adopted^25^ to more precisely etching stop above the M-layer by use of selective chemical etching process. Figures 2b and 3b show the calculated electric fields along the horizontal directions (BB’) at the bottommost M-layers under V_br_ for devices A and B, respectively. As expected, we can clearly see that there is a reduction in the electric field from 305 (610) to 100 (120) kV/cm at the sidewall of the bottommost M-layer in device A (B). As a result, our proposed APD has lower leakage current, better E-field confinement, and edge breakdown suppression.

### Experimental results

Figure 4a–c represents the measured bias-dependent dark current and photocurrent of our three devices namely A, A′ and B when subjected to different optical pumping powers at an optical wavelength of 1.55 µm. Here, device A′ has the exact same epi-layer structure as device A but the charge layer doping density has been tweaked (2.5E+17 vs. 2E+17/cm^3^)) to have a lager reverse bias punch through voltage (V_pt_) (− 16 vs. − 9 V). The purpose of device A′ is to investigate the influence of charge layer doping density on the device high-power performance, which will be discussed latter in Fig. 8. The measured breakdown voltages (V_br_) of device A, A′, and B are around − 43, − 41, and − 51 V, respectively. Here, the V_br_ values are defined as the corresponding bias voltages for 10 µA leakage current of each device. Comparison shows that the values of the M- and absorption layer thickness of device A are close to those of device B, but the V_br_ and V_pt_ exhibited are both smaller than those of device B. This result is in contrast to that for the traditional APD where a lower V_br_ always comes with a higher V_pt_ due to the increase of the charge layer doping density, which implies that the effective critical field in the M-region of device A is much less than that in B (305 vs. 610 kV/cm), as indicated in Figs. 2 and 3. The theoretical maximum unit gain responsivity for 2 µm-thick In_0.53_Ga_0.47_As absorption layer will be close to 1 A/W at the 1.55 µm wavelength. The photo-absorption constant considered for the In_0.53_Ga_0.47_As layer at this wavelength is approximately 0.8 µm^−1^^26^ when considering zero-coupling loss and single-pass light injection in the absorption layer for both devices. The gain versus bias voltage for devices A, A′ and B under different optical pumping powers (1 to 250 µW) is shown in Fig. 4d–f. We can clearly observe that device A exhibits a much larger maximum gain and a higher responsivity under 0.95 V_br_ than device B due to more pronounced multiplication process induced by the additional M-layer (3 vs. 2) in device A. A sudden decrease in the operation gain at higher pumping power is observed in both the devices which can be explained by the SCS effect induced by the photo-generation of holes in the thick and undoped In_0.53_Ga_0.47_As absorption layer, which lowers the net E-field and multiplication gain in the M-layer^14^. Due to the stronger E-field inside In_0.53_Ga_0.47_As absorption layer of device A, as illustrated in Figs. 2 and 3, the decrease of gain with the increase of pumping power becomes less serious than that of device B, indicative of a superior high-power performance. The high-power performance of both devices will be discussed in detail in the following sections.

**Figure 5:**
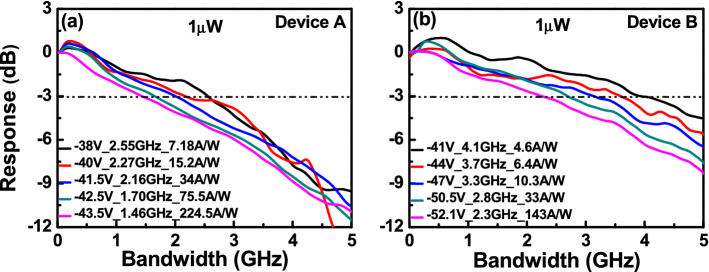
Measured bias dependent O-E frequency responses under 1 µW optical pumping power of: (**a**) Device A. (**b**) Device B.

**Figure 6:**
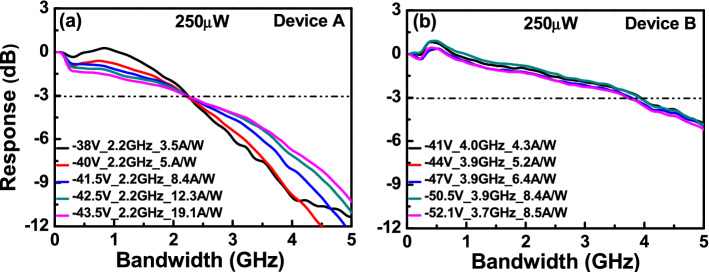
Measured bias dependent O-E frequency responses under 250 µW optical pumping power of: (**a**) Device A. (**b**) Device B.

Figures 5 and 6 illustrates the bias-dependent O-E frequency responses of devices A and B, measured at a wavelength of 1.55 µm under low (1 µW) and high (250 µW) optical pumping powers. For both low and high optical pumping powers, device B has a larger 3-dB O-E bandwidth than does device A, under the same operational responsivity. This is due to it having fewer M-layers (2 vs. 3) and a shorter avalanche delay time, as expected. Moreover, under a low (1 µW) optical pumping power as shown in Fig. 5, there is only a slight degradation in the bandwidth (from 1.7 (2.8) to 1.46 (2.3) GHz for device A (B)) when the bias voltage of each device is close to their respective V_br_. This contradicts from the typical behavior of most APDs, which exhibits a significant decrease in O-E bandwidth as the operation gain increases due to an increase in the avalanche induced delay time. The use of cascade avalanche processes, as explained above in our multiple M-layer design, has resolved this trade-off between operation gain and speed. Similarly, regardless of changes in the reverse bias voltages, the measured 3-dB O-E bandwidths of devices A and B are maintained at about 2.2 and 3.9 GHz, respectively, under high optical pumping power, as shown in Fig. 6. Such stable high-speed performance can be accredited to a serious reduction in the value of the operation gain, which becomes considerably less sensitive to the reverse bias voltage under high-power operation, as illustrated in Fig. 4^14^.

**Figure 7:**
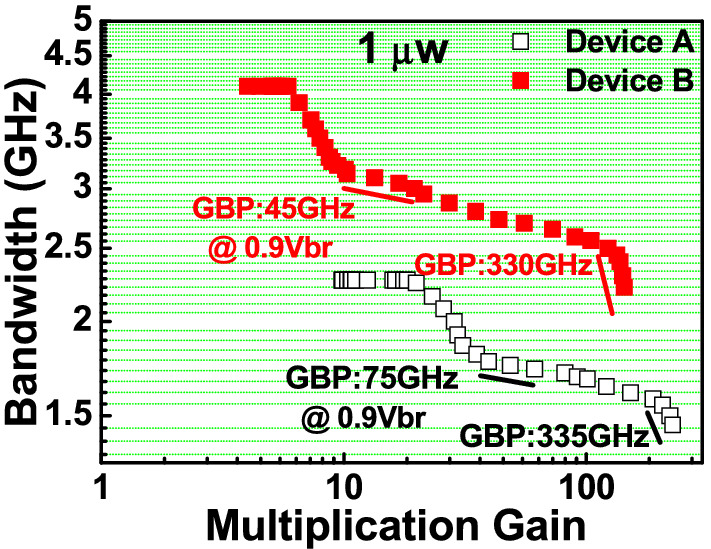
Measured 3-dB O-E bandwidths versus multiplication gain of the demonstrated APD at low (1 µW) optical pumping power for. (**a**) Device A. (**b**) Device B.

Figure 7 depicts the 3-dB O-E bandwidth versus operation gains of devices A and B when measured with a low (1 µW) optical input power. Here, the gain-bandwidth product (GBP) values are defined at around the operation window (G: ~ 20 at 0.9V_br_) and extremely high-gain (> 100) regions, respectively. At 0.9 V_br_ operation point, device A exhibits higher GBP (75 vs 45) than that of device B due to the more pronounced multiplication process induced by the additional M-layer (3 vs. 2) inside device A. In addition, both the devices have similar GBP values of 330 GHz under extremely high gain operations. This implies that the maximum intrinsic speed performance in both the devices are close due to their close values of absorption- and M-layers thickness as discussed in Fig. 1^27^. The cascade avalanche process facilitated by the multiple M-layer design is accountable for this high GB product. In addition, our device exhibits a wider operation window when compared to the gain-bandwidth curves of traditional APDs, that usually shows a monotonic decrease in bandwidth with an increase in multiplication gain^1,2^. This implies that a constant O-E bandwidth (~ 1.7 (A) and ~ 2.8 (B) GHz) can be retained over a wide range of operation gains (from ~ 20 to ~ 100). Such consistent performance will be useful in FMCW LiDAR applications, which will be described in greater depth later. As discussed above, in the FMCW LiDAR receiver, the high saturation current characteristic, in addition to the high-responsivity performance, plays a significant role.

**Figure 8:**
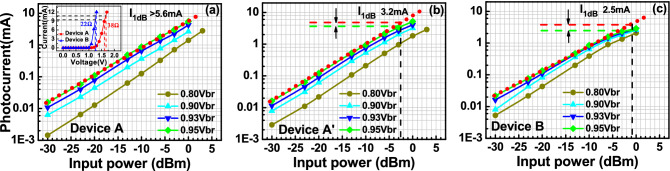
Measured DC output photocurrent versus input optical input power for. (**a**) Device A. (**b**) Device A′. (**c**) Device B. Inset to (**a**) shows the measured forward bias I-V curves of devices A and B.

**Figure 9:**
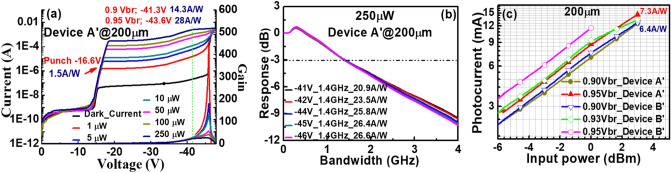
Device A′ with a 200 µm active window diameter showing. (**a**) Measured dark current, photocurrent, and operation gain versus bias voltages under different optical pumping powers. (**b**) Bias dependent O-E frequency responses under 250 µW optical pumping power. (**c**) DC output photocurrent vs. input optical power at different bias voltages of devices A′ and B′ with the same active window diameter of 200 µm.

**Figure 10:**
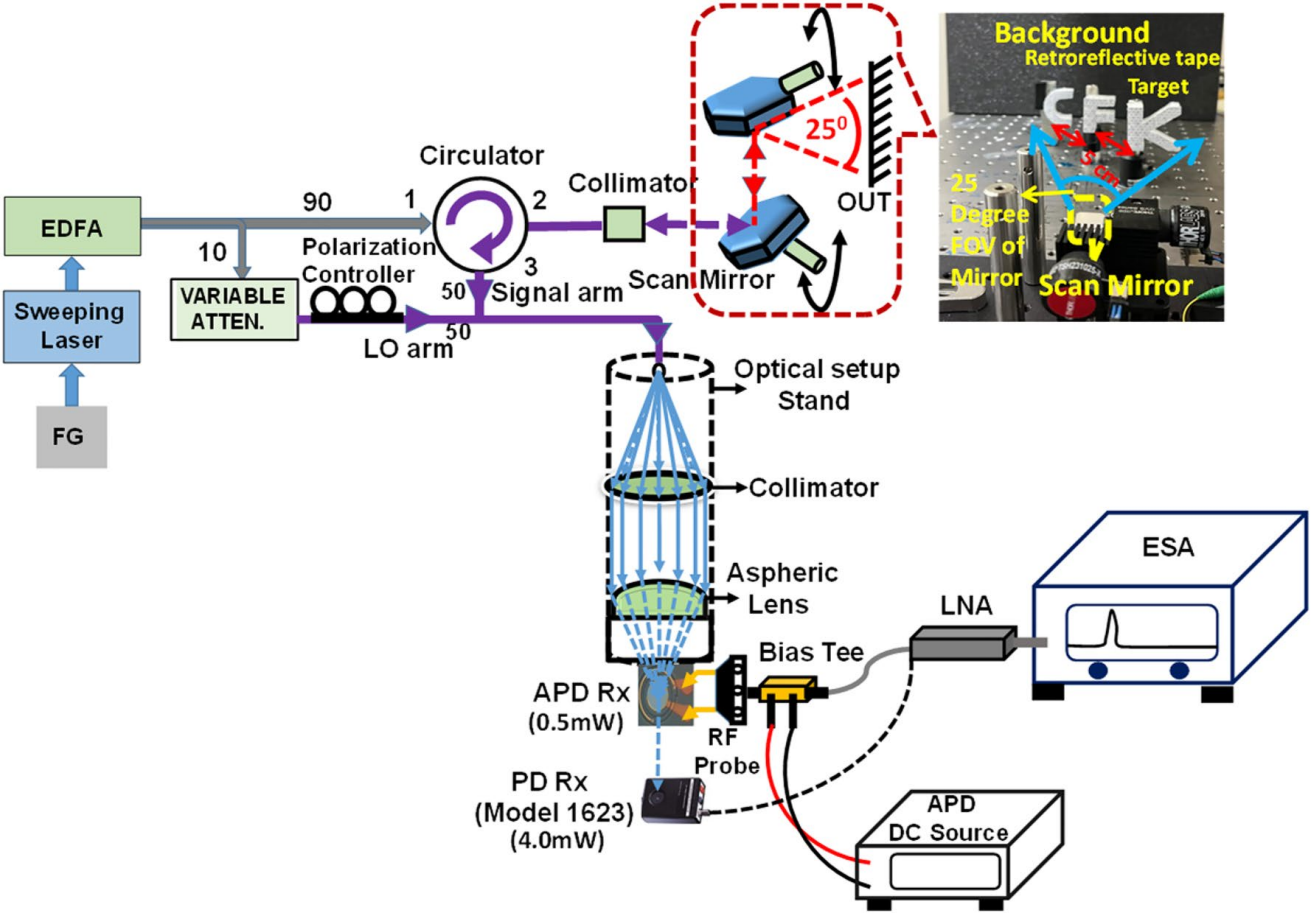
Schematic representation of the demonstrated FMCW LiDAR system with APD Rx (Device A and Device B) and p-i-n PD Rx.

**Figure 11:**
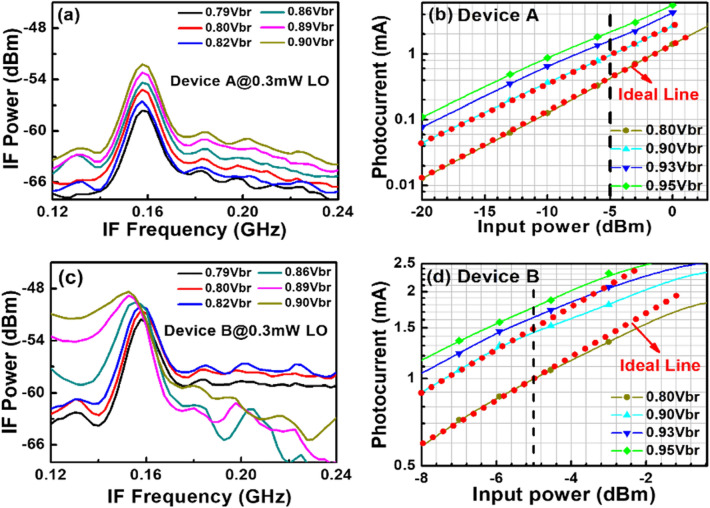
(**a**), (**c**) shows measured bias dependent IF spectra at a fixed 0.3mW LO power for Device A and Device B. (**b**), (**d**) shows the close-up versions of Fig. 8a,c, respectively.

**Figure 12:**
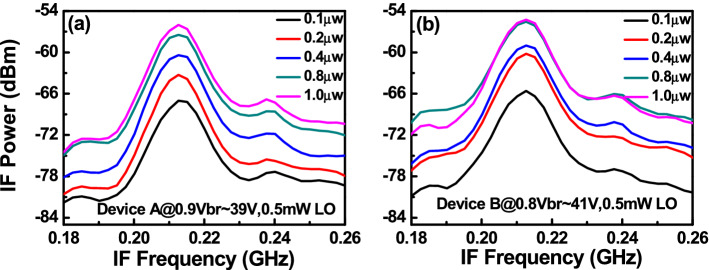
Measured IF spectra under fixed 0.5mW LO power with different received powers for. (**a**) Device A at 0.9V_br_. (**b**) Device B at 0.8V_br_.

**Figure 13:**
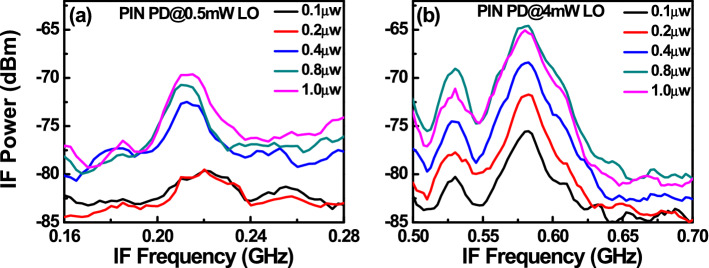
Measured IF spectra of p-i-n PD module with different received powers under (**a**) 0.5mW (**b**) 4mW optical LO power.

**Figure 14:**
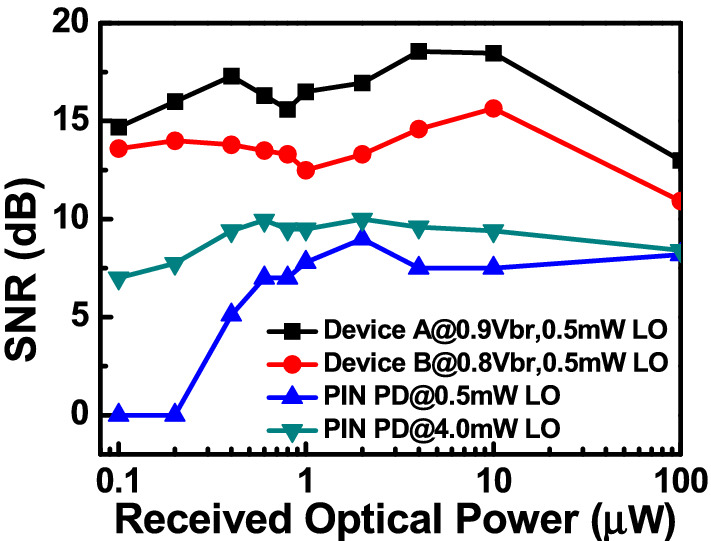
Measured SNR vs. received optical power of different receivers under optimal conditions.

**Figure 15:**
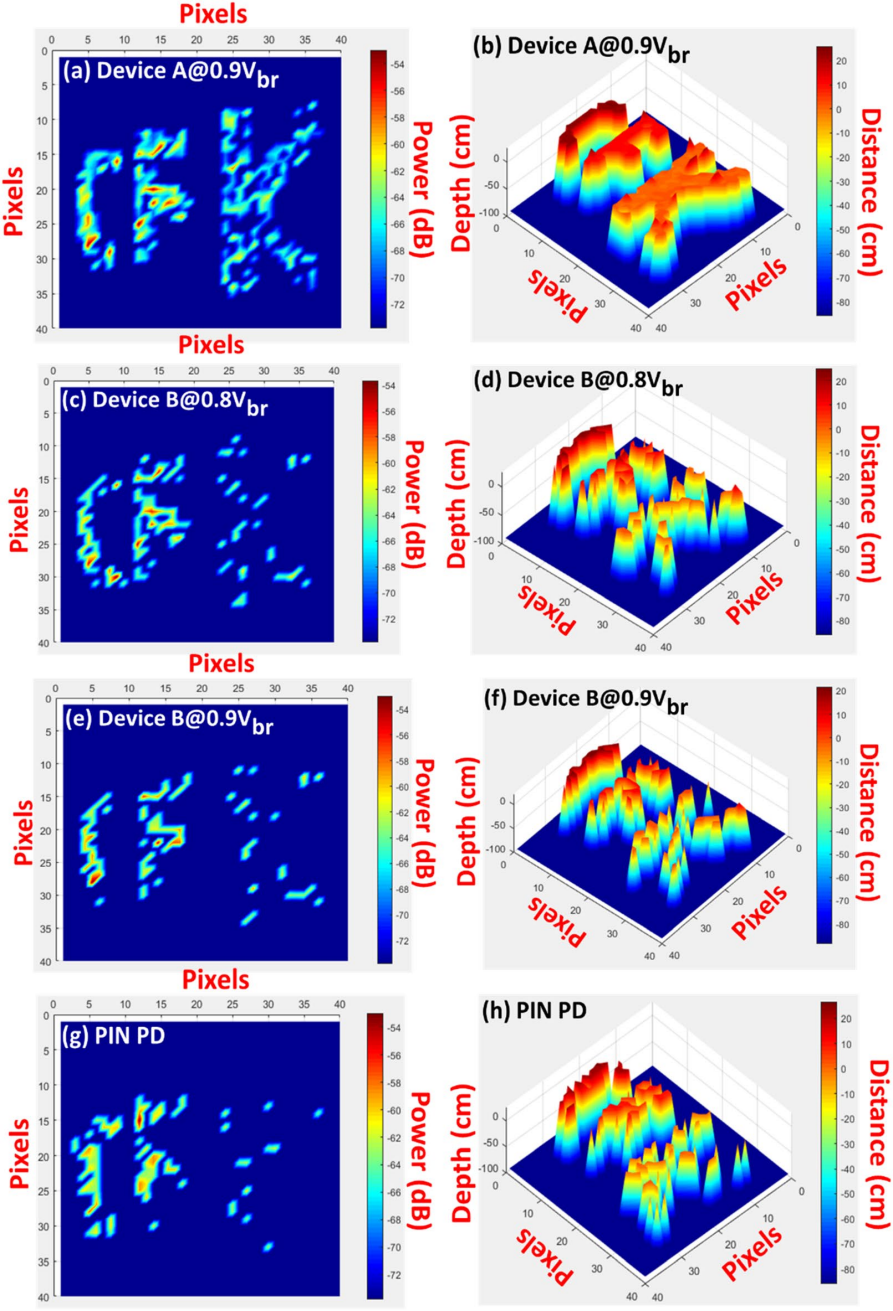
(**a**,**c**,**e**,**g**) shows captured LiDAR images corresponding to the measured IF power of each pixel from Device A at 0.9V_br_, Device B at 0.8V_br_, Device B at 0.9V_br_ and the p-i-n PD. (**b**,**d**,**f**,**h**) shows captured LiDAR 3D images based on the depth and distance information of each pixel from Device A at 0.9V_br_, Device B at 0.8V_br_, Device B at 0.9V_br_ and the p-i-n PD.

Figure 8 depicts the measured dc output photocurrent versus the input optical power for devices A, A′, and B. These three devices have the same active window diameter of 60 µm. As can be seen, device A has a higher 1 dB saturation current (I_1dB_: > 5.6 mA vs. 3.2 (A′) and 2.5 (B) mA @ 0.95 V_br_) than those of the other two devices. This is because it has the smallest V_pt_ and largest E-field inside the thick In_0.53_Ga_0.47_As absorber layer of these three devices, as discussed in Figs. 2 and 3. In contrast to traditional APDs, the lower V_pt_ of device A does not lead to an increase in the operation voltage (~ 0.9 V_br_) which would avoid more serious device heating under high output photocurrents. Except for the heat generated from the IV product of the device under operation, ohmic heating, which is generated from the parasitic resistance of our APDs may be an issue affecting the saturation current performances. The inset to Fig. 8a shows the measured forward bias I-V curves of devices A and B. We can clearly see that device A has a slightly higher turn-on voltage, larger differential resistances, and more serious ohmic heating than does device B, due to its thicker overall depletion layer. This result clearly indicates that the ohmic heating effect does not actually play a significant role in the measured saturation current of both devices. The higher I1dB in device A is mainly due to its larger internal E-field in the absorber, as has already been discussed. Furthermore, the output photocurrent of device A does not show significant saturation at a bias of 0.95 V_br_ and can reach nearly 6 mA. Under such high output current conditions, the corresponding responsivity can be as high as 6 A/W. With its high responsivity at high output photocurrents, our demonstrated APD can be used in a coherent FMCW LiDAR system, allowing for a higher S/N ratio and a lower optical power budget than traditional p-i-n PD-based receivers, as will be discussed further. In order to pursue a higher saturation current with our device, it is necessary to further enlarge the active window diameter to lower the current and heat densities under high-power operation. Figure 9a illustrates the measured bias-dependent dark current, photocurrent, and operation gain of device A′ with a 200 µm active window diameter, under various optical pumping powers at 1.55 µm optical wavelength. Compared with the devices with a smaller active window size, shown in Fig. 4, the maximum operation gain can be further boosted from 200 to around 500. One possible reason for this phenomenon is the existence of less surface state induced leakage current density from the edge of APD when the active mesa size becomes larger. The measured bias-dependent O-E frequency responses of this device at high (250 µW) optical pumping powers are shown in Fig. 9b. We can clearly see that the 3-dB O-E bandwidth is pinned at 1.4 GHz, close to that obtained with device B (1.2 GHz) which has the same active window diameter and measured at the same optical pumping power^21^. This result contrasts with the measurement results shown in Figs. 5 and 6 and it can be attributed to the net O-E bandwidths of both devices being limited by the RC-bandwidth instead of internal carrier response time for the case of an APD with such a large window size. For fairness of comparison, Fig. 9c depicts the measured dc output photocurrent versus the input optical power for devices A′ and B, which have the same active window diameter of 200 µm. We can clearly see that the saturation current of device A′ exceeds 14.6 mA under 0.95 V_br_ and is limited by device failure. The corresponding responsivity is as high as 7.3 A/W. On the other hand, device B shows a significant saturation of output photocurrent at around 12 mA under 0.93 V_br_ operation. When the bias voltage is further increased to 0.95 V_br_, device failures under a lower optical pumping power can be observed with no improvement in the maximum output current (~ 12 mA). The excellent high-power performance of device A′ compared to device B is mainly due to its lower operation voltage and V_br_ (− 45 (A) vs. − 53 V (B)) and less device heating with a comparable high responsivity. This measurement results support the conclusion that it would be advantageous to use our novel multiple M-layer design in an APD to facilitate a lower effective critical field for avalanche breakdown. Figure 10 shows the fiber-based FMCW LiDAR system with mechanical scanning mirrors^28^ that was built to test the feasibility of our APDs for coherent FMCW LiDAR applications. The modulated output light from the sweeping laser source is amplified with EDFA and then split into a signal and a local-oscillator (LO) channel with a 90/10 fiber splitter. The interested reader can refer to our previous work^21,22^ for more information on our wavelength sweeping laser module and the optical setup working principle. In our FMCW LiDAR system, we used two types of receivers: our home-made APD’s and a commercially available p-i-n PD module (Newport 1623). The photomixed down-converted IF signal from the receivers is sent to an RF spectrum analyzer for analysis. The FMCW LiDAR system is tested using letter shaped Styrofoam targets (K, F, and C) placed at specified distances. The targets are wrapped with retroreflective tape, as seen in Fig. 10. Figure 11a,c shows the measured bias dependent IF spectra of devices A and B, respectively, obtained under a fixed optical local oscillator (LO) and signal powers of 0.3 mW and 0.1 µW, respectively. Here, both devices have the same active window diameter as 60 µm for comparison. As can be seen, the output IF tones from device B exhibit significant broadening when the operation voltage is over 0.82 V_br_. On the other hand, with device A one can maintain a Gaussian like IF spectrum from low to high bias voltages (0.8 to 0.9 V_br_). Figure 11b,d shows close-up version of Fig. 8a,c, respectively. We can clearly see that the distorted IF spectra in device B can be attributed to the saturation phenomenon of the output photocurrent under 0.3mW (~ − 5dBm) optical pumping power. This becomes more pronounced when the reverse bias operation voltage goes higher. In contrast to device B, there is no significant saturation of photocurrent under different operation conditions for device A, which leads to an unchanging IF spectrum for bias voltages from low to high. Figure 12a,b depicts the measured IF spectra for devices A and B, obtained with a fixed optical LO power (0.5 mW), optimized operation voltages (device A: 0.9 V_br_; device B: 0.8 V_br_) and different received optical signal powers. For comparison, a commercially available p-i-n PD module with a built-in 50 Ω load (Newport 1623) is used as a receiver in our FMCW LiDAR test bed. With a 3-dB O-E bandwidth at nearly 0.6 GHz, it has a responsivity of 1 A/W, which is the same as the unit gain responsivity of our demonstrated APDs. Figure 13a,b shows the measured IF spectra of this PD module under low (0.5 mW) and high (4 mW) optical LO power, respectively, and different received optical signal powers. The corresponding signal-to-noise (SNR) ratio of the measured IF spectra from these three devices are given in Fig. 14. The SNR is defined here as the difference in power level between the IF tone's peak and its corresponding noise floor. We can clearly see that device A has the best SNR performance among these three devices due to its superior responsivity and output saturation photocurrent, compared to those of device B and the p-i-n PD module. Moreover, in contrast to the traditional p-i-n PD based receiver, our demonstrated APDs has a much larger SNR (15 vs. 7 dB) with much less optical LO power required (0.5 vs. 4 mW). This advantage can greatly increase the optical power budget in the modern FMCW LiDAR module, where the output optical power from the wavelength sweeping light source is always limited. Images of the object under tests (OUTs; K, F, and C) acquired in our well-established FMCW LiDAR system using devices A and B and the p-i-n PD module as the receiver are shown in Fig. 15a–h.

To create these images, we use down-converted IF frequencies and power in each pixel. In this case, the scanning mirror's free space optical power output is around 12 mW, while the collected weak optical power is in the sub- µW range. Based on the measurement results previously stated in Figs. 11, 12, 13, the optical LO pumping power and bias voltages applied to the APDs has been optimized. Here we choose the averaged values of the measured IF frequency from each pixels of letter K as our reference frequency. The color contrast between the letters K, F, and C in Fig. 15b,d,f,h represents the measured round trip LiDAR distances.

**Figure 16:**
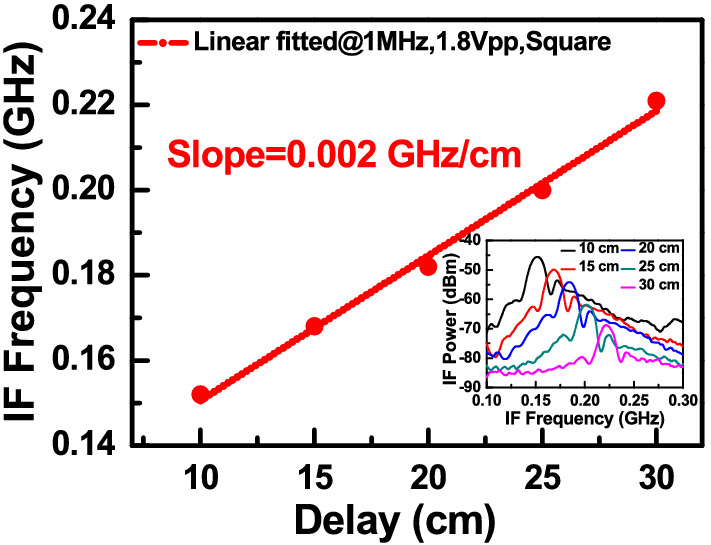
The IF beating frequency versus different delay line distances. Inset picture shows the corresponding IF spectra for different delays.

**Figure 17:**
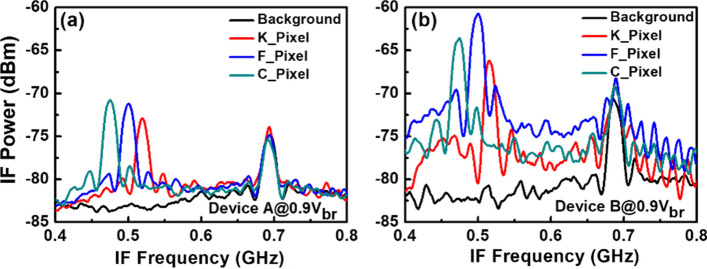
IF spectra of a single pixel captured from the background of the K, F and C shaped targets using APDs for. (**a**) Device A at 0.9V_br_. (**b**) Device B at 0.9V_br_.

We can clearly see that letter “K” locates at reference plane as discussed above and the LiDAR measured distances between “F” and “C” is around 10 and 20 cm, respectively. These values are in accordance with the real distances between these three targets, as shown in Fig. 10. Figure 16 depicts the beating intermediate frequency (IF) versus different delay line distances (time) for our sweeping laser source under the driving conditions specified in the figures. The corresponding output IF spectra for device A are shown in the inset. Changing the distance between the OUT and the scanning mirror changes the length of the delay line. As can be seen, a linear relationship can be obtained between the delay time (distance) and the beating frequency, which is considered necessary for a high performance FMCW LiDAR system. The slope of the trace and the difference in the IF frequency between the pixels of the targets can be used to measure the distance information in these captured images. Figure 17a,b represents the typical measured IF spectra of pixels from the targets (K, F, and C) and the background at the optimized conditions for device A (0.9 V_br_) and device B at a higher bias (0.9 V_br_). Here, the background signal is induced by parasitic reflection between port 3 of the circulator and the coupling optics (collimators or fiber connectors) used for feeding the light onto the APD based receiving end. As can be seen, at the optimized conditions of devices A, it gives less noisy IF spectra with a lower noise floor compared to those obtained from device B at a 0.9 V_br_ bias. This can be explained based on Fig. 11, while measuring a single pixel in the beating setup, the saturation of the output photocurrent in device B under 0.9 V_br_ bias leads to the broadening of the IF linewidth and the increase of its noise level. Consequently, such saturation phenomenon in device B, results in the degraded SNR of its output signal and blurrier 3-D image with fewer pixels captured in Fig. 15f than the image shown in Fig. 15d. In summary, device A clearly demonstrates the best quality of captured 3-D images among the 3 devices, which can be attributed to its excellent performance in terms of high responsivity and high saturation current mentioned above. The results are in accordance with the SNR shown in Fig. 14.

## Conclusion

In this work, we demonstrate a novel APD design to overcome the fundamental trade-off between the responsivity and saturation current performance which occurs in traditional APDs used for frequency modulated continuous wave (FMCW) LiDAR and coherent communication applications. By adopting multiple In_0.52_Al_0.48_As based multiplication (M-) layers with a stepped electric (E-) field inside, the effective critical field can be effectively reduced due to the more pronounced avalanche process which occurs within the multiple M-regions than those of dual M-layer reference sample. We can thus allocate a stronger E-field within the thick absorption layer of the APD with a smaller operation voltage. This in turn leads to a less serious space-charge screening effect and less device heating under high output photocurrents. Compared with a dual M-layer reference sample with the same active window size (60 µm), the demonstrated APD exhibit both smaller V_pt_ and V_br_, higher responsivity (19.6 vs. 13.5 A/W), larger maximum gain (230 vs. 130), and higher 1-dB saturation current (> 5.6 vs. 2.5 mA) under 0.95 V_br_ operation. Moreover, with a further increase in the device active window diameter to 200 µm with a low output current density, the demonstrated device structure still sustains a superior saturation current performance to that of device B (> 14.6 vs. 12.8 mA) due to the smaller operation voltage (V_br_) and less serious device heating of device A. In the coherent FMCW LiDAR test bed, this novel APD exhibits a larger signal-to-noise ratio in each pixel and much better quality of constructed 3-D images than those of obtained with the reference dual M-layer sample and high-performance commercial p-i-n PD modules, with much less optical local-oscillator (LO) power required (0.5 vs. 4 mW). These results strongly support that such novel APDs can further enhance the sensitivity of the next generation FMCW LiDAR and coherent communication systems.

## Data Availability

The experimental datasets and the analysis discussed in the paper and any additional datasets that support the plots within this paper and other finding of this study are available from the corresponding author upon reasonable request.

## References

[1] Campbell JC (2004). Recent advances in avalanche photodiodes. IEEE J. Sel. Top. Quantum Electron..

[2] Nada M, Yamada Y, Matsuzaki H (2018). Responsivity-bandwidth limit of avalanche photodiodes: Toward further ethernet systems. IEEE J. Sel. Top. Quantum Electron..

[3] Daniel R, Almog R, Ron A, Belkin S, Diamand YS (2008). Modeling and measurement of a whole-cell bioluminescent biosensor based on a single photon avalanche diode. Biosens. Bioelectron..

[4] Morimoto K (2020). Megapixel time-gated SPAD image sensor for 2D and 3D imaging applications. Optica.

[5] Kuzmenko K (2020). 3D LIDAR imaging using Ge-on-Si single–photon avalanche diode detectors. Opt. Express.

[6] Ferraro MS (2019). Position sensing and high bandwidth data communication using impact ionization engineered APD arrays. IEEE Photonics Technol. Lett..

[7] Yi X (2019). Extremely low excess noise and high sensitivity AlAs_0.56_Sb_0.44_ avalanche photodiodes. Nat. Photonics.

[8] Ceccarelli F, Acconcia G, Gulinatti A, Ghioni M, Rech I, Osellame R (2021). Recent advances and future perspectives of single-photon avalanche diodes for quantum photonics applications. Biosens. Bioelectron. Adv. Quantum Technol..

[9] Kardynał BE, Yuan ZL, Shields AJ (2008). An avalanche-photodiode-based photon-number-resolving detector. Nat. Photonics.

[10] Arrazola JM (2021). Quantum circuits with many photons on a programmable nanophotonic chip. Nature.

[11] Chitnis D, Collins S (2014). A SPAD-based photon detecting system for optical communications. J. Lightwave Technol..

[12] Abedin MN, Refaat TF, Sulima OV, Singh UN (2004). AlGaAsSb-InGaAsSb HPTs with high optical gain and wide dynamic range. IEEE Trans. on Electron Devices.

[13] Sze, S. M. *Physics of Semiconductor Devices*, ch. 13 (Wiley, 1981).

[14] Zhao HY (2019). High-speed avalanche photodiodes with wide dynamic range performance. J. Lightwave Technol..

[15] Nada M, Yamada Y, Matsuzaki H (2017). A high-linearity avalanche photodiodes with a dual-carrier injection structure. IEEE Photon. Technol. Lett..

[16] Runge P (2018). Waveguide Integrated Balanced Photodetectors for Coherent Receivers. IEEE J. Sel. Top. Quantum Electron..

[17] Adany P, Allen C, Hui R (2009). Chirped LiDAR using simplified homodyne detection. J. Lightwave Technol..

[18] Zhang X (2022). A large-scale microelectromechanical-systems-based silicon photonics LiDAR. Nature.

[19] Martin A (2018). Photonic integrated circuit-based FMCW coherent LiDAR. J. Lightwave Technol..

[20] Isaac BJ, Song B, Pinna S, Coldren LA, Klamkin J (2019). Indium phosphide photonic integrated circuit transceiver for FMCW LiDAR. IEEE J. Sel. Top. Quantum Electron..

[21] Ahmad Z (2021). High-Power and high-responsivity avalanche photodiodes for self-heterodyne FMCW LiDAR system applications. IEEE Access.

[22] Ahmad Z (2022). Avalanche photodiodes with dual multiplication layers and ultra-high responsivity-bandwidth products for FMCW LiDAR system applications. IEEE J. Sel. Top. Quantum Electron..

[23] Goh YL, Ng JS, Tan CH, Ng WK, David JPR (2005). Excess noise measurement in In_0.53_Ga_0.47_As. IEEE Photon. Technol. Lett..

[24] Saleh MA (2001). Impact-ionization and noise characteristics of thin III-V avalanche photodiodes. IEEE Trans. Electron Devices.

[25] Naseem (2022). Avalanche photodiodes with composite charge-layers for low dark current, high-speed, and high-power performance. IEEE J. Sel. Top. Quantum Electron..

[26] Naseem (2019). The enhancement in speed and responsivity of uni-traveling carrier photodiodes with GaAs_0.5_Sb_0.5_/In_0.53_Ga_0.47_As type-II hybrid absorbers. Opt. Express.

[27] Kinsey GS, Campbell JC, Dentai AG (2001). Waveguide avalanche photodiode operating at 1.55 μm with a gain-bandwidth product of 320 GHz. IEEE Photon. Technol. Lett..

[28] Barber ZW, Dahl JR, Sharpe TL, Erkmen BI (2013). Shot noise statistics and information theory of sensitivity limits in frequency-modulated continuous-wave ladar. J. Opt. Soc. Am. A.

